# Pyrimidine containing furanose derivative having antifungal, antioxidant, and anticancer activity

**DOI:** 10.1186/s13588-014-0003-0

**Published:** 2014-07-27

**Authors:** Rupesh Dudhe, Pramod Kumar Sharma, Prabhakar Kumar Verma

**Affiliations:** 1 Uttarakhand Technical University, Dehradun, 284007 Uttarakhand India; 2 Department of Pharmacy, SMAS, Galgotias University, Greater Noida, 201306 Uttar Pradesh India; 3 Department of Pharmaceutical Sciences, M. D. University, Rohtak, 124001 Haryana India

**Keywords:** Pyrimidine, Anticancer, Antifungal, Antioxidant

## Abstract

**Background:**

A series of 6-(substituted aldehyde)-3,4-dihydro-1-(tetrahydro-3,4-dihydroxy-5-(hydroxymethyl) furan-2-yl)-4-phenylpyrimidine-2(1H)-one derivative (**6A-6P**) was synthesized from the 6-(substituted aldehyde)-4-phenylpyrimidine-2(1H)-one derivative **(5A-5P)** through following reaction mechanisms Claisen-Schmidt, Cyclization, and Satos fusion. The structures of the synthesized compounds were elucidated by I.R.,^1^H-NMR, elemental analysis, and mass spectroscopic techniques.

**Result:**

The synthesized compounds were screened for *in vitro* antifungal activity at 25, 50, 100, and 200 μg/ml concentrations. Among them, compounds **6P, 6D,** and **6M** exhibited significant antifungal activity that was carried out by cup plate method against fungal strain which was collected from IMTECH Chandigarh, India, against standard drug fluconazole. Compounds have been further evaluated by measuring zone of inhibition and percent inhibition. The synthesized compounds were screened for *in vitro* antioxidant activity using the DPPH assay, based on the AAI and antioxidant activity unit (AAU), using a combination relation between DPPH concentration and absorbance. The antioxidant strength of compounds was compared against ascorbic acid. Among them, compounds **6K, 6F, 6E, 6G, 6H,** and **6M** exhibited significant antioxidant activity and **6J** have less active compound. The data of these synthesized compounds were submitted to the National Institute of Health, USA, under the drug discovery program of National Cancer Institute (NCI) and screened for anticancer activity at a single high dose (10^−5^ M) in full NCI 60 cell lines. The selected compounds have shown potent significant anticancer activity in the NCI 60 cell line screening.

**Conclusion:**

A new series of pyrimidine analogues that contain furanose moiety were synthesized by Satos fusion and characterized. The synthesized compounds screened for their *in vitro* antioxidant, antifungal activity, as well as anticancer activity given by the derivative which has chloro, methoxy, nitro, and chloro substitution having furanose contain pyrimidine derivative that showed the most potent activity.

**Electronic supplementary material:**

The online version of this article (doi:10.1186/s13588-014-0003-0) contains supplementary material, which is available to authorized users.

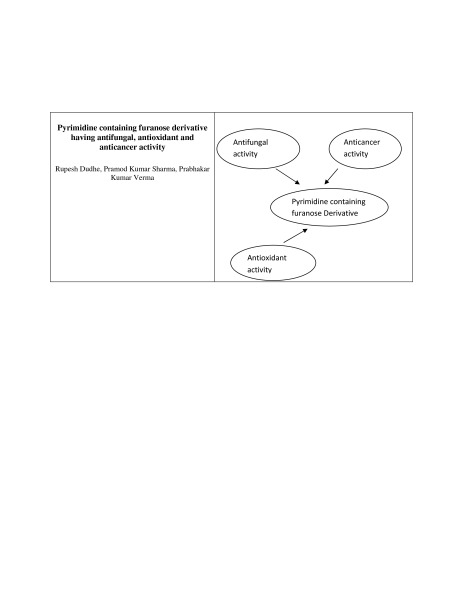

## Background

Pyrimidine is a six-member heterocyclic compound that contains two nitrogen atoms at positions 1 and 3. Pyrimidines, being an integral part of DNA and RNA impart to diverse pharmacological properties as effective bactericide and fungicides [1], nitrogen containing heterocyclic ring such as pyrimidine is a promising structural moiety for drug designing. Pyrimidine derivatives form a component in a number of useful drugs and are associated with many biological and therapeutically activities [2]. Condensed furanose pyrimidine derivatives have been reported as antioxidant [3], antimicrobial [4], analgesic [5], antiviral [6], anti-inflammatory [7], anti-HIV [8], antitubercular [9], antitumor [10], antineoplastic [11], and antimalarial [12]. The condensed furanose pyrimidine derivative has the power to accept free radical during different abovementioned diseases due to the presence of NH and OH molecule in ring.

Free radicals are well known for playing a dual role in our body, deleterious as well as beneficial. It includes metabolic pathway for its generation [13]. It mainly explores the formation and the scavenging of free radicals, as well as the damage caused by free radicals in biological system. Oxidative stress in our body occurs due to excessive generation of free radical and reduced level of antioxidant, but at low concentration, these radicals performs normal physiological functions of body. Scientific evidence suggests that antioxidants reduce the risk for chronic diseases including cancer and heart disease [14].

**Figure 1:**
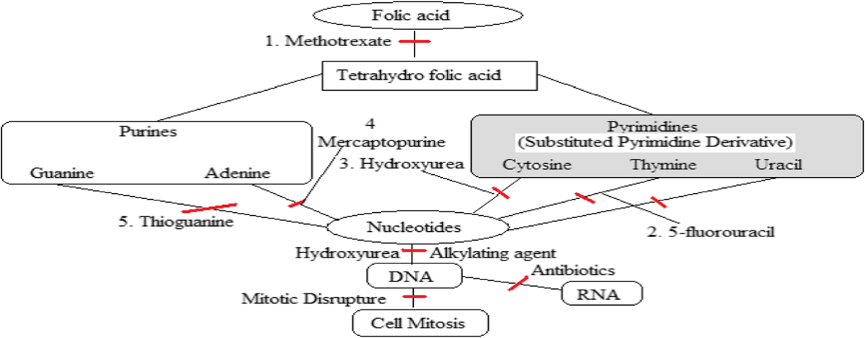
**Mechanism of anticancer compound.**

About 13% deaths of human beings throughout the world are caused by cancer, which is characterized by uncontrolled cell growth, metastasis, and invasion [13]. Although the risk of cancer increases with age, people of all ages, even fetuses, can be affected by the disease. The most occurring fatal cancers are lung, stomach, liver, colon, and breast cancer. Therefore, continuous search for new anticancer agents should be an active work in the research field at various laboratories. Among potential anticancer agents, heterocyclic compounds represent an outstanding type of anticancer drug moiety (Figure 1).

**Figure 2:**
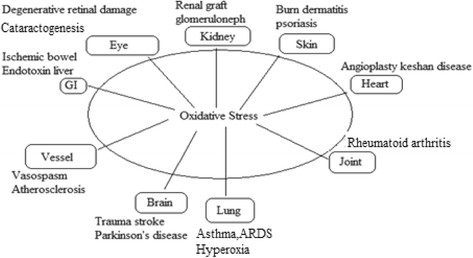
**Effect of free radical in the body parts.**

Free radical may be defined as the atoms, molecules, or ions with unpaired electrons in an open shell configuration. Sometime, free radicals may contain positive, negative, or zero charge [15]. Free radicals play an important role in combustion, atmospheric chemistry, polymerization, plasma chemistry, and many other chemical processes [16]. The large generation of free radicals particularly reactive oxygen species and their high activity plays an important role in the progression of great number of pathological disturbances such as inflammation [17], atherosclerosis [18], cancer [19], parkinsonism [20], and Alzheimer's disease [21]. Inflammation is mostly caused through excessive generation of free radical in the body (Figure 2).

## Methods

### Chemistry

All the reagents and solvents used were laboratory grade and obtained from the supplier (Sigma-Aldrich, St. Louis, MO, USA; CDH, Beijing, China; and Rankem, Faridabad, India) or recrystallized/redistilled as necessary. The purity of compounds synthesized, commercial reagents used, and monitor of reaction was done by thin layer chromatography (TLC) plates (silica gel G). Two solvent systems: toluene/ethyl acetate/formic acid (5:4:1) and ethyl acetate/n-hexane (3:7) were used to run TLC. The spots were located under iodine vapors and UV light. The melting points of the products were determined by open capillaries method and are uncorrected. Infrared (IR) spectra (KBr) were recorded on FTIR spectrophotometer (Shimadzu FTIR 8400 S, 4,000 to 400 cm^−1^, Kyoto, Japan). The elemental analysis was carried out using Heraus CHN rapid analyzer (Hanau, Germany) [1].H-NMR spectra were recorded on a JEOL AL300 FTNMR 300 MHz spectrometer (Akishima-shi, Japan) in dimethyl sulfoxide (DMSO) using TMS as an internal standard, with [1] H resonance frequency of 300 MHz chemical shift values are expressed in *δ* ppm. The activity was performed on instrument UV-visible spectrophotometer UV-1800 Pharmaspec Shimadzu.

### Screening of compounds

#### Antifungal activity

**Figure 3:**
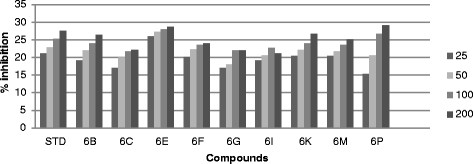
**Antifungal activity of synthesized compound against** ***Candida tropicalis*** **(MTCC-230).**

**Figure 4:**
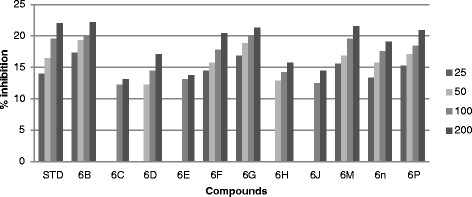
**Antifungal activity of synthesized compound against** ***Candida glabrata*** **(MTCC-3019).**

**Figure 5:**
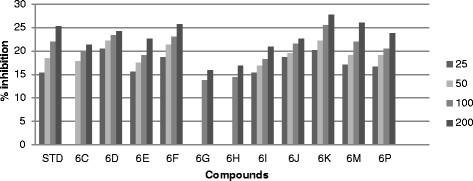
**Antifungal activity of synthesized compound against** ***Candida inconspicua*** **(MTCC-1074).**

**Figure 6:**
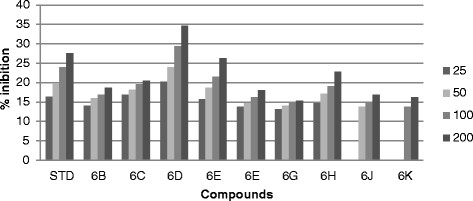
**Antifungal activity of synthesized compound against** ***Candida albicans*** **(MTCC-227).**

**Figure 7:**
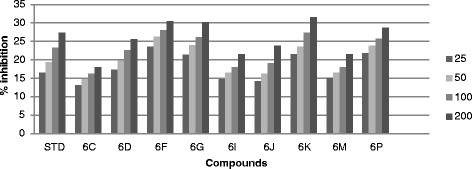
**Antifungal activity of synthesized compound against** ***Candida viswanathii*** **(MTCC-1629).**

All the synthesized compound were screened *in vitro* for their antifungal activity *Candida inconspicua* (Microbial Type culture collection (MTCC)-1074, American Type Culture Collection (ATCC)-16783), *Candida viswanathii* (MTCC-1629, ATCC-22981), *Candida albicans* (MTCC-227, ATCC-10231), *Candida tropicalis* (MTCC-230, ATCC-20336), *Candida glabrata* (MTCC-3019, ATCC-90030) against standard drug fluconazole [19]. The incubation time was 48 h at 37°C for fungal strain. All the screened compounds were found to possess moderate to good antifungal activity. The cup plate test was performed using agar medium and dextrose agar medium, and the medium was autoclaved at 15 lbs pressure (121°C) for 15 min then immediately cooled to 50°C to 55°C in a water bath after removing it from autoclave. The cooled medium was poured into sterile petri plates to a uniform depth of 4 mm or 25 ml in a 90-mm plate. Once the medium had solidified, then the culture was inoculated on the medium by a sterile swab that was dipped into the fungus suspension or inoculated with 1 ml of the organism suspension. Sterillized 9-mm cork borer was used to make agar wells, than placed 25, 50, 100 and 200 μg/ml diluted test compound as well as standard compound were placed into each wells and DMSO as a control. The plate were inoculate for 48 h at 37°C for fungal strain and measure zone of inhibition in mm and the percentage (%) of inhibition was calculated by using the formula [17] (Tables 1, 2, 3, 4 and 5) (Figures 3, 4, 5, 6 and 7).

1 $$\%\;\mathrm{of}\;\mathrm{inhibition}=\frac{\mathrm{Diameter}\;\mathrm{of}\;\mathrm{the}\;\mathrm{inhibition}\;\mathrm{zone}\;\mathrm{in}\;\mathrm{mm}}{\mathrm{Diameter}\;\mathrm{of}\;\mathrm{the}\;\mathrm{petri}\;\mathrm{plates}\;\mathrm{in}\;\mathrm{mm}\;\left(90\right)}$$

**Table 1:** **Antifungal activity for MTCC-230 strain**

Compounds	% Inhibition at μg/ml MTCC-230			
	25	50	100	200
Standard	21.11	22.89	25.33	27.56
6B	19.11	22	24	26.44
6C	17.11	20	21.7	22.24
6E	26	27.33	28	28.66
6F	20.22	22.26	23.55	24
6G	17.11	18	22	22.04
6I	19.11	20.66	22.66	21.2
6K	20.4	22.22	24	26.66
6M	20.44	21.7	23.55	25.2
6P	15.33	20.66	26.66	29.1

**Table 2:** **Antifungal activity for MTCC-3019**

Compounds	% Inhibition at μg/ml MTCC-3019			
	25	50	100	200
Standard	14.00	16.44	19.56	22.00
6B	17.33	19.33	20	22.22
6C	0	0	12.22	13.11
6D	0	12.22	14.44	17.11
6E	0	0	13.11	13.77
6F	14.44	15.77	17.77	20.44
6G	16.88	18.88	20.04	21.33
6H	0	12.88	14.22	15.77
6J	0	0	12.44	14.44
6M	15.55	16.88	19.55	21.55
6n	13.33	15.77	17.55	19.11
6P	15.3	17.07	18.44	20.88

**Table 3:** **Antifungal activity for MTCC-1074**

Compounds	% Inhibition at μg/ml MTCC-1074			
	25	50	100	200
Standard	15.33	18.44	22.00	25.33
6C	0	17.77	20	21.33
6D	20.44	22.24	23.33	24.22
6E	15.55	17.55	19.11	22.66
6F	18.66	21.33	23.11	25.77
6G	0	0	13.77	15.88
6H	0	0	14.44	16.88
6I	15.33	16.88	18.22	20.97
6J	18.66	19.55	21.55	22.66
6K	20.22	22.22	25.55	27.77
6M	17.11	19.11	22	26
6P	16.66	19.11	20.44	23.77

**Table 4:** **Antifungal activity for MTCC-227**

Compounds	% Inhibition at μg/ml MTCC-227			
	25	50	100	200
Standard	16.44	19.33	23.33	27.33
6B	14	16	16.88	18.66
6C	16.88	18.22	19.55	20.44
6D	20.2	24	29.33	34.66
6E	15.77	18.66	21.55	26.22
6E	13.77	14.66	16.22	18
6G	13.11	14	14.66	15.33
6H	14.66	17.11	19.11	22.8
6J	0	13.77	15.11	16.88
6K	0	0	13.77	16.22

**Table 5:** **Antifungal activity for MTCC-1629**

Compounds	% Inhibition at μg/ml MTCC-1629			
	25	50	100	200
Standard	16.44	19.33	23.33	27.33
6C	13.11	14.66	16.22	18
6D	17.33	19.77	22.6	25.53
6F	23.55	26.22	28	30.44
6G	21.33	24	26.11	30.22
6I	14.66	16.44	18	21.55
6J	14.22	16.22	19.11	23.77
6K	21.55	23.55	27.33	31.55
6M	15.11	16.44	18	21.55
6P	21.77	23.77	25.77	28.66

#### Free radical scavenging method by DPPH assay

**Figure 8:**
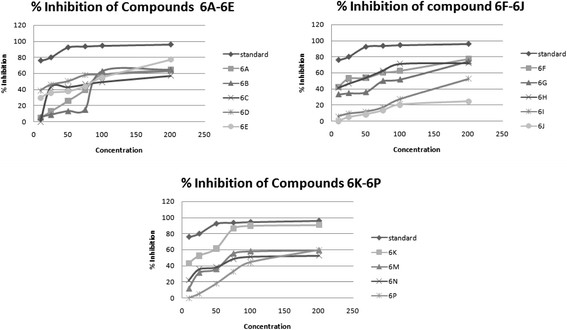
**Percent inhibition for antioxidant activity of pyrimidine derivative.**

**Scheme 1:**
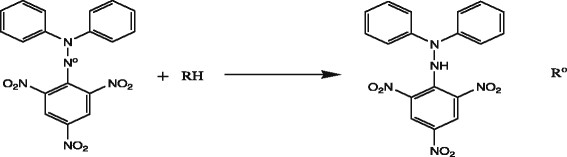
**Reaction of DPPH with compound having proton.**

Various concentrations of test compound 10 to 200 μg/ml were prepared in the methanol and 1 ml of each concentration was added to 1 ml of 0.1 mM solution of DPPH [3]. The mixture was shaken vigorously and allowed to stand for 30 min in dark place; absorbance at 517 nm was determined by UV spectrometer, and the percentage scavenging activity was calculated. A blank solution of DPPH was prepared, and ascorbic acid was used as reference compound. All the compounds were tested and analyzed by their absorbance. The equation used to measure free radical scavenging is as follows (Equation 1, Figure 8, Scheme 1):

A lower value of mean inhibitory concentration shows a higher free radical scavenging activity.

2 $$\%\;\mathrm{Scavenging}=\frac{\mathrm{Absorbance}\;\mathrm{of}\;\mathrm{control}-\mathrm{Absorbance}\;\mathrm{of}\;\mathrm{test}\;\mathrm{compounds}/\mathrm{Std}.}{\mathrm{Absorbance}\;\mathrm{of}\;\mathrm{control}}\times 100$$

### AAU equation

**Figure 9:**
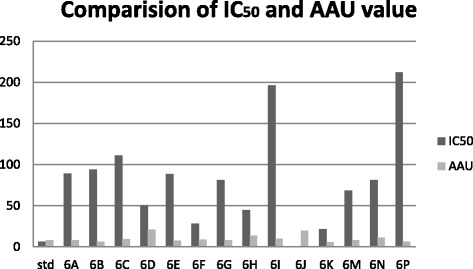
**AAU and IC50 value.**

The free radical scavenging fitting curve equation (*y* = BX + D) when combined with theoretical value of DPPH concentration and absorbance (*y* = KX) to assume the index antioxidant activity unit (AAU), it is defined as ‘one mole of DPPH free radical was completely scavenged to consume amount (mole number) of the scavenger’. The lower the value of AAU, the stronger the antioxidant ability of compound (Table 6) (Figure 9).

3 $$\mathrm{AAU}=394.32\times \frac{R}{B\times C\times \mathrm{Mr}},$$

where *R* = solution volume ratio of sample to solution volume of DPPH for each sample, *B* = slope of fitting equation of free radical scavenging ratio, *C* = initial concentration of DPPH solution observed, and Mr = molecular weight of sample.

**Table 6:** **AAU and IC50 value of synthesized pyrimidine derivatives**

Sample number	Compound code	Slop	IC_50_value μg/ml	***r*** ^2^	AAU
1	6A	0.566	89	0.704	8.076
2	6B	0.835	94	0.322	8.13
3	6C	0.753	111	0.35	6.47
4	6D	0.764	50	0.257	9.43
5	6E	0.76	88.5	0.117	20.76
6	6F	0.912	28.5	0.312	7.69
7	6G	0.763	81	0.27	8.9
8	6H	0.891	45	0.293	8.15
9	6I	0.767	196	0.164	13.56
10	6J	0.984	0	0.249	10.05
11	6K	0.872	21.5	0.126	19.86
12	6M	0.775	68.5	0.368	5.9
13	6N	0.819	81.45	0.279	8.37
14	6P	0.761	212	0.209	11.19

#### Anticancer screening

Pharmacological evaluation of the anticancer activity was performed on the compounds inconvertibly selected by the National Institute of Health, Bethesda, USA, under the drug discovery program of National Cancer Institute (NCI) [13]. All the finally synthesized 20 compounds have been registered on its website, and from those, only 13 compounds have been selected. All the selected compounds have been given a unique NCI number [19].

### Methodology of the *in vitro* cancer screening

The human tumor cell lines of the cancer screening panel are grown in RPMI 1640 medium containing 5% fetal bovine serum and 2 μL glutamine. The cells are inoculated into 96-well microtiter plates in 100 μL at plating densities ranging from 5,000 to 40,000 cells/well depending on the doubling time of individual cell lines. After cell inoculation, the microtiter plates are incubated at 37°C in the presence of 5% CO_2_, 95% air, and 100% relative humidity for 24 h prior to addition of experimental drugs. After 24 h, two plates of each cell line are fixed *in situ* with TCA to represent a measurement of the cell population for each cell line at the time of drug addition (Tz). Experimental drugs are solubilized in dimethyl sulfoxide at desired final maximum test concentration and stored frozen prior to use. At the time of drug addition, an aliquot of frozen concentrate is thawed and diluted to twice the desired final maximum test concentration with complete medium containing 50 μg/ml gentamicin. Additional 4-, 10-fold, or ½ log serial dilutions are made to provide a total of five drug concentrations plus control. Aliquots of 100 μl of these different drug dilutions are added to the appropriate microtiter wells already containing 100 μl of medium, resulting in the required final drug concentrations.

After the following drug addition, the plates are incubated for an additional 48 h at 37°C, 5% CO_2_, 95% air, and 100% relative humidity. For adherent cells, the assay is terminated by the addition of cold TCA. Cells are fixed *in situ* by the gentle addition of 50 μl of cold 50% (*w*/*v*) TCA (final concentration, 10% TCA) and incubated for 60 min at 4°C. The supernatant is discarded, and the plates are washed five times with tap water and air dried. Sulforhodamine B (SRB) solution (100 μl) at 0.4% (*w*/*v*) in 1% acetic acid is added to each well, and plates are incubated for 10 min at room temperature. After staining, unbound dye is removed by washing five times with 1% acetic acid and the plates are air dried. Bound stain is subsequently solubilized with 10 mM trizma base, and the absorbance is read on an automated plate reader at a wavelength of 515 nm. For suspension cells, the methodology is the same except that the assay is terminated by fixing settled cells at the bottom of the wells by gently adding 50 μl of 80% TCA (final concentration, 16% TCA). Using the seven absorbance measurements (time zero, (Tz), control growth, (C), and test growth in the presence of drug at the five concentration levels (Ti)), the percentage growth is calculated at each of the drug concentrations levels. Percentage growth inhibition is calculated as:

4 $$\left[\left(\mathrm{Ti}-\mathrm{Tz}\right)/\left(C-\mathrm{Tz}\right)\right]\;\times \;100\;\mathrm{for}\;\mathrm{concentrations}\;\mathrm{for}\;\mathrm{which}\;\mathrm{Ti}>/=\mathrm{Tz}$$

5 $$\left[\left(\mathrm{Ti}-\mathrm{Tz}\right)/\mathrm{Tz}\right]\;\times \;100\;\mathrm{for}\;\mathrm{concentrations}\;\mathrm{for}\;\mathrm{which}\;\mathrm{Ti}<\mathrm{Tz}.$$

**Figure 10:**
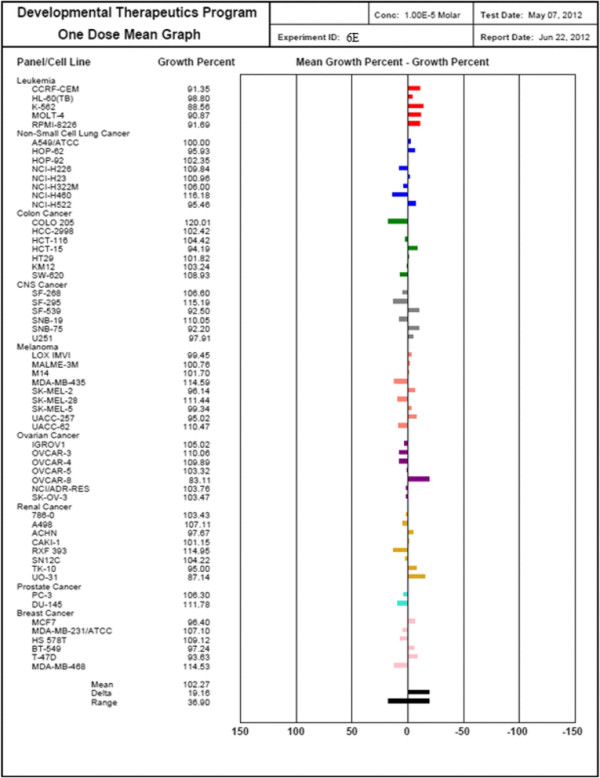
**Growth percent of cancer cells of compound 6E.**

**Figure 11:**
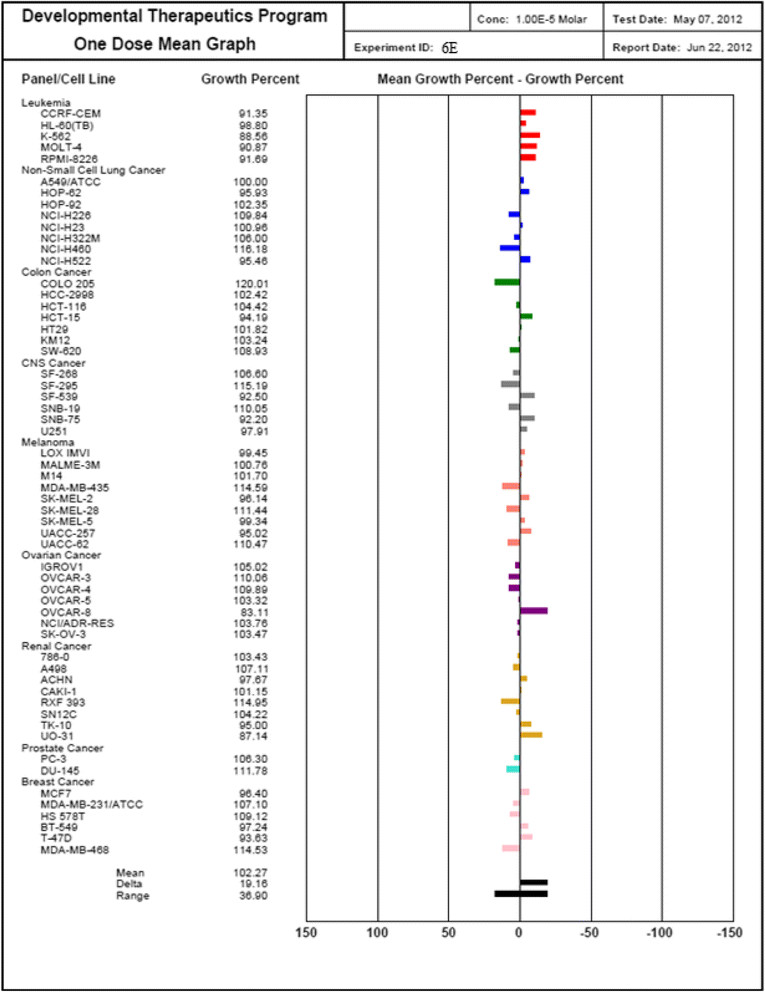
**Growth percent of cancer cells of compound 6N.**

**Figure 12:**
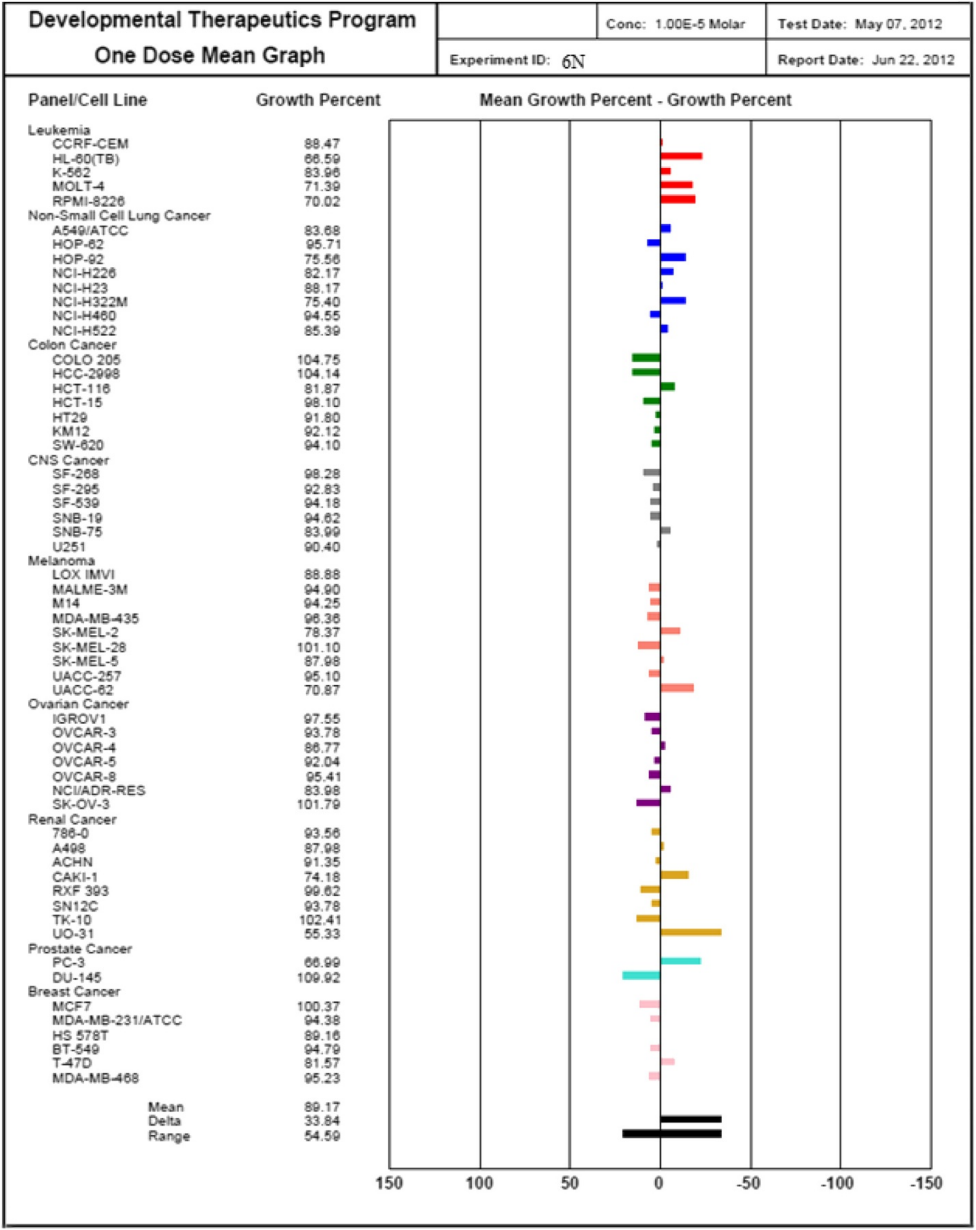
**Growth percent of cancer cells of 6P.**

**Figure 13:**
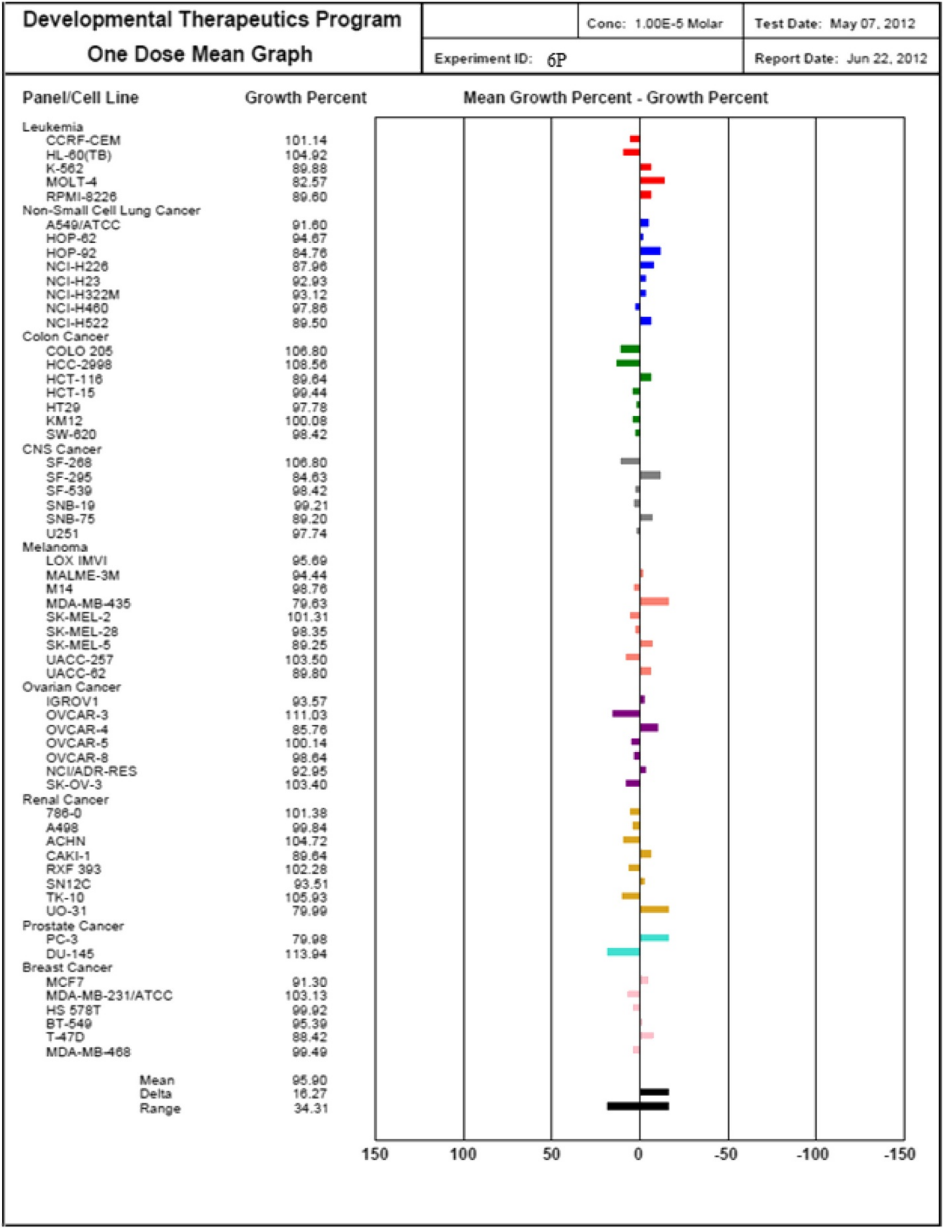
**Growth percent of cancer cells of 6C.**

Three dose response parameters are calculated for each experimental agent. Growth inhibition of 50% (GI50) is calculated from [(Ti − Tz)/(C − Tz)] × 100 = 50, which is the drug concentration resulting in a 50% reduction in the net protein increase (as measured by SRB staining) in control cells during the drug incubation. The drug concentration resulting in total growth inhibition (TGI) is calculated from Ti = Tz. The LC50 (concentration of drug resulting in a 50% reduction in the measured protein at the end of the drug treatment as compared to that at the beginning) indicating a net loss of cells following treatment is calculated from [(Ti − Tz)/Tz] × 100 = −50. Values are calculated for each of these three parameters if the level of activity is reached; however, if the effect is not reached or is exceeded, the value for that parameter is expressed as greater or less than the maximum or minimum concentration tested. The compounds which reduce the growth of any one of the cell lines by 32% or less are passed on for further evaluation in the full panel of 60 cell lines (Table 7) (Figures 10, 11, 12 and 13).

**Table 7:** **Growth percentage of synthesized compound against cancer cell line**

Code number	Mean	Growth percent of cancer cells								
6E	102.27	92.25	103.34	105.00	102.40	103.21	102.66	101.33	109.04	103.00
6N	89.17	76.08	85.07	95.26	92.38	89.75	93.04	87.27	88.45	92.58
6P	95.90	93.62	91.55	100.10	96.00	94.52	97.92	97.16	96.96	96.27
6C	100.69	95.66	96.75	103.51	96.14	102.20	102.64	101.08	112.87	102.25

### General procedures for the synthesis of compounds

#### Synthesis of compounds (3A-3P)

**Scheme 2:**
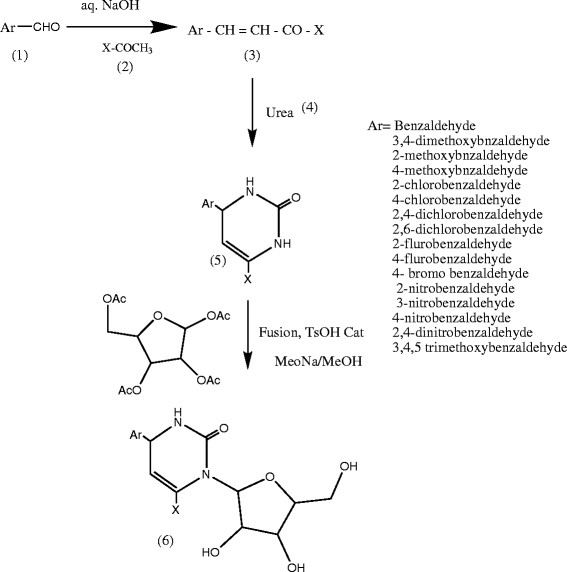
**Reaction scheme.**

Equimolar portions of the appropriately substituted aromatic aldehyde (10 mmol) and acetophenone (10 mmol) were dissolved in approximately in 15 ml of ethanol. The mixture was allowed to stir for several minutes at 5°C to 10°C. Then, 10 ml of 40% aq. NaOH solution was added dropwise to the reaction mixture in the conical flask. The reaction mixture then allowed stirring at room temperature for 4 h on stirrer and precipitate is allowed to stand overnight in refrigerator. Precipitate is formed which is collected by filtration and repeatedly washed with distilled water and finally recrystallized in ethanol. The solvent system was used for the TLC ethyl acetate/n-hexane (3:7) (Scheme 2).

***Synthesis of 3-phenyl-1-phenylprop-2-en-1-one (chalcone) (3A):*** It was obtained by reaction of acetophenon with benzaldehyde. Molecular formula, C_17_H_16_O_3_; molecular weight, 208; m.p., 58°C to 60°C; Rf. value (ethyl acetate/n-hexane, 3:7), 0.78; IR (KBr, cm^−1^), 3,001.03(C-H stretch.), 1,660.60(conj. C = C), 1,604.66(Ar. C = C), 1,288.36(Ar. C-O), 688.54(Ar. C-H bend).

***Synthesis of 3-(3,4-dimethoxyphenyl)-1-phenylprop-2-en-1-one (3B):*** It was obtained by reaction of acetophenon (2) with 3,4-dimethoxy benzaldehyde. Molecular formula, C_17_H_16_O_3_; molecular weight, 268; m.p., 76°C to 78°C; Rf. value (ethyl acetate/n-hexane, 3:7), 0.80; I.R. (KBr, cm^−1^), 2,939.31(C-H str.), 1,654.81(conj. C = C), 1,587.31(Ar. C = C), 1,255.57(Ar. C-O), 698.18(Ar. C-H bend).

***Synthesis of 3-(2-methoxyphenyl)-1-phenylprop-2-en-1-one (3C):*** It was obtained by reaction of acetophenon with 2-methoxy benzaldehyde. Molecular formula, C_16_H_14_O_2_; molecular weight, 238; m. p., 52°C to 54°C; Rf. value (ethyl acetate/n-hexane, 3:7), 0.72; I.R. (KBr, cm^−1^), 1,670.44(conj. C = C), 1,602.74(Ar. C = C), 1,249.79(Ar. C-O), 692.40(Ar. C-H bend).

***Synthesis of 3-(4-methoxyphenyl)-1-phenylprop-2-en-1-one (3D):*** It was obtained by reaction of acetophenon with 4-methoxy benzaldehyde. Molecular formula, C_16_H_14_O_2_; molecular weight, 238; m. p., 72°C to 74°C; Rf. value (Ethyl acetate/n-hexane, 3:7), 0.76; I.R. (KBr, cm^−1^), 1,656.74(conj. C = C), 1,600.81(Ar. C = C), 1,213.14(Ar. C-O), 688.54(Ar. C-H bend.).

***Synthesis of 3-(2-chlorophenyl)-1-phenylprop-2-en-1-one (3E):*** It was obtained by reaction of acetophenon with 2-chloro benzaldehyde. Molecular formula, C_15_H_11_ClO; molecular weight, 242; m. p., 55°C to 57°C; Rf. value (ethyl acetate/n-hexane, 3:7), 0.78; I.R. (KBr, cm^−1^), 2,956.67(C-H str.), 1,660.60(conj. C = C), 1,577.66(Ar. C = C), 1,249.79(Ar. C-O), 752.19 (C-Cl), 692.40(Ar. C-H bend.).

***Synthesis of 3-(4-chlorophenyl)-1-phenylprop-2-en-1-one (3F):*** It was obtained by reaction of acetophenon- with 4-chloro benzaldehyde. Molecular formula, C_15_H_11_ClO; molecular weight, 242; m. p., 80°C to 84°C; Rf. value (ethyl acetate/n-hexane, 3:7), 0.75; I.R. (KBr, cm^−1^), 2,918.10(C-H, str.), 1,658.67(conj. C = C), 1,602.74 (Ar. C = C), 1,217.00(Ar. C-O), 775.33 (C-Cl), 690.47(Ar. C-H bend.).

***Synthesis of 3-(2, 4-dichlorophenyl)-1-phenylprop-2-en-1-one (3G):*** It was obtained by reaction of acetophenon with 2,4-dichlorobenzaldehyde. Molecular formula, C_15_H_10_Cl_2_O; molecular weight, 277; m. p., 70°C to 72°C; Rf. value (ethyl acetate/n-hexane, 3:7), 0.72; I.R. (KBr, cm^−1^), 2,958.60(C-H str.), 1,662.52(conj. C = C), 1,608.52(Ar. C = C), 1,286.36(Ar. C-O), 713.61 (C-Cl), 684.68(Ar. C-H bend.).

***Synthesis of 3-(2, 6-dichlorophenyl)-1-phenylprop-2-en-1-one (3H):*** It was obtained by reaction of acetophenon with 2,6-dichlorobenzaldehyde. Molecular formula, C_15_H_10_Cl_2_O; molecular weight, 277; m. p., 76°C to 78°C; Rf. value (ethyl acetate/n-hexane, 3:7), 0.74; I.R. (KBr, cm^−1^), 3,082.04(C-H str.), 1,660.60(conj. C = C), 1,612.38(Ar. C = C), 1,265.22(Ar. C-O), 719.40 (C-Cl), 696.25(Ar. C-H bend.).

***Synthesis of 3-(2-fluorophenyl)-1-phenylprop-2-en-1-one (3I):*** It was obtained by reaction of acetophenon with 2-fluorobenzaldehyde. Molecular formula, C_15_H_11_FO; molecular weight, 226; m. p., 38°C to 40°C; Rf. value (ethyl acetate/n-hexane, 3:7), 0.78; I.R. (KBr, cm^−1^), 3,028.03(C-H str.), 1,641.31(conj. C = C), 1,612.38(Ar. C = C), 1,384.79 (C-F), 1,244.36(Ar. C-O), 686.61(Ar. C-H bend.).

***Synthesis of 3-(4-fluorophenyl)-1-phenylprop-2-en-1-one (3J):*** It was obtained by reaction of acetophenon with 4-flurobenzaldehyde. Molecular formula, C_15_H_11_FO; molecular weight, 226; m. p., 40°C to 42°C; Rf. value (ethyl acetate/n-hexane, 3:7), 0.80; I.R. (KBr, cm^−1^), 1,696.60(conj. C = C), 1,609.59(Ar. C = C), 1,382.87 (C-F), 1,213.14(Ar. C-O), 688.54(Ar. C-H bend).

***Synthesis of 3-(4-bromophenyl)-1-phenylprop-2-en-1-one (3K):*** It was obtained by reaction of acetophenon with 4-bromobenzaldehyde. Molecular formula, C_15_H_11_BrO; molecular weight, 287; m. p., 112°C to 114°C; Rf. value (ethyl acetate/n-hexane, 3:7), 0.72; I.R. (KBr, cm^−1^), 3,056.96(C-H str.), 1,658.67(conj. C = C), 1,608.52(Ar. C = C), 1,332.72(Ar. C-O), 688.54(Ar. C-H bend), 532.32(C-Br).

***Synthesis of 3-(2-nitrophenyl)-1-phenylprop-2-en-1-one (3L):*** It was obtained by reaction of acetophenon with 2-nitrobenzaldehyde. Molecular formula, C_15_H_11_NO_3_; molecular weight, 253; m. p., 78°C to 80°C; Rf. value (ethyl acetate/n-hexane, 3:7), 0.68; I.R. (KBr, cm^−1^), 3,006.82(C-H str.), 1,631.67(conj. C = C), 1,382.87 (Ar-NO_2_), 1,276.79(Ar. C-O), 686.88(Ar. C-H bend).

***Synthesis of 3-(3-nitrophenyl)-1-phenylprop-2-en-1-one (3M):*** It was obtained by reaction of acetophenon with 3-nitrobenzaldehyde. Molecular formula, C_15_H_11_NO_3_; molecular weight, 253; m. p., 68°C to 70°C; Rf. value (ethyl acetate/n-hexane, 3:7), 0.66; I.R. (KBr, cm^−1^), 2,918.10(C-H str.), 1,662.52(conj. C = C), 1,608.52(Ar. C = C), 1,529.45(Ar-NO_2_), 1,218.93(Ar. C-O), 655.75(Ar. C-H bend.).

***Synthesis of 3-(4-nitrophenyl)-1-phenylprop-2-en-1-one (3N):*** It was obtained by reaction of acetophenon with 4-nitrobenzaldehyde. Molecular formula, C_15_H_11_NO_3_; molecular weight, 253; m. p., 62°C to 74°C; Rf. value (ethyl acetate/n-hexane, 3:7), 0.74; I.R. (KBr, cm^−1^), 2,968.24(C-H str.), 1,629.74(conj. C = C), 1,595.02(Ar. C = C), 1,384.79 (Ar-NO_2_), 1,218.93(Ar. C-O), 684.68(Ar. C-H bend).

***Synthesis of 3-(2,4-dinitrophenyl)-1-phenylprop-2-en-1-one (3O):*** It was obtained by reaction of acetophenon with 2,4-dinitrobenzaldehyde. Molecular formula, C_15_H_10_N_2_O_5_; molecular weight, 253; m. p., 92°C to 94°C; Rf. value (ethyl acetate/n-hexane, 3:7), 0.78; I.R. (KBr, cm^−1^), 2,918.10(C-H str.), 1,631.67(conj. C = C), 1,531.37 (Ar-C-NO_2_), 1,384.79 (Ar-NO_2_), 1,218.93(Ar. C-O), 688.54(Ar. C-H bend.).

***Synthesis of 3-(3, 4, 5-trimethoxyphenyl)-1-phenylprop-2-en-1-one (3P):*** It was obtained by reaction of acetophenon with 3,4,5-trimethoxybenzaldehyde. Molecular formula, C_18_H_18_O_4_; molecular weight, 298.33; Rf. value (ethyl acetate/n-hexane; 3:7), 0.78; I.R. (KBr, cm^−1^) 2,908.45(C-H str.), 1,662.52(conj. C = C), 1,232.43(Ar. C-O), 665.54(Ar. C-H bend.).

### Synthesis of compounds (5A-5P): general procedure

A mixture of compound, i.e., substituted chalcone (0.01 M) (3A-3P), add 0.01 M of NaOH and urea (0.01 M) was refluxed in ethanol for 8 to 10 h after completion of reaction. The content was concentrated and poured into cold water. The product so obtained was washed with water repeatedly and recrystallized in ethanol.

***Synthesis of 3,4-dihydro-4,6-diphenylpyrimidin-2(1H)-one (5A):*** It was obtained by reaction of compound 3-phenyl-1-phenylprop-2-en-1-one(chalcone) (3A) (0.01 M), add 0.01 M of NaOH and urea (0.01 M). Molecular formula, C_16_H_14_N_2_O; molecular weight, 250.2; m. p., 111°C to 113°C.; Rf. value (toluene/ethyl acetate/formic acid, 5:4:1), 0.64; I.R. value (KBr, cm^−1^), 3,319.26(N-H), 3,001.03(Ar C-H), 1,625.88(C = O), 1,579.59(C = C), 854.53(C-H bend.).

***Synthesis of 3,4-dihydro-4-(2, 4-dimethoxyphenyl)-6-phenylpyrimidin-2(1H)-one (5B):*** It was obtained by reaction of compound 3-(2,4-dimethoxyphenyl)-1-phenylprop-2-en-1-one (3B) (0.01 M), add 0.01 M of NaOH and urea (0.01 M). Molecular formula, C_18_H_18_N_2_O3; molecular weight, 310.32; m. p., 121°C to 123°C.; Rf. value (toluene/ethyl acetate/formic acid, 5:4:1), 0.68; I.R. value (KBr, cm^−1^), 3,137.97(N-H), 3,008.75(Ar C-H), 1,670.24(C = O), 1,544.88(C = C), 842.83(C-H bend.).

***Synthesis of 3,4-dihydro-4-(2-methoxyphenyl)-6-phenylpyrimidin-2(1H)-one (5C):*** It was obtained by reaction of compound 3-(2-methoxyphenyl)-1-phenylprop-2-en-1-one (3C) (0.01 M), add 0.01 M of NaOH and urea (0.01 M). Molecular formula, C_17_H_16_N_2_O_2_; molecular weight, 380.32; m. p., 132°C to 134°C.; Rf. value (toluene/ethyl acetate/formic acid, 5:4:1), 0.76; I.R. value, 3,440.77(N-H), 2,916.17(C-H str.), 1,645.17(C = O), 1,596.95(N-H bend.), 838.98(C-H bend.).

***Synthesis of 3,4-dihydro-4-(4-methoxyphenyl)-6-phenylpyrimidin-2(1H)-one (5D):*** It was obtained by reaction of compound 3-(4-methoxyphenyl)-1-phenylprop-2-en-1-one (3D) (0.01 M), add 0.01 M of NaOH and urea (0.01 M). Molecular formula, C_17_H_16_N_2_O_2_; molecular weight, 380.32; Rf. value (toluene/ethyl acetate/formic acid, 5:4:1), 0.78; I.R. value, 3,470.67(N-H), 2,903.63(C-H str.), 1,684.70(C = O), 1,584.41(N-H bend.), 815.83(C-H bend.).

***Synthesis of 4-(2-chlorophenyl)-3,4-dihydro-6-phenylpyrimidin-2(1H)-one (5E):*** It was obtained by reaction of compound 3-(2-chlorophenyl)-1-phenylprop-2-en-1-one (3E) (0.01 M), add 0.01 M of NaOH and urea (0.01 M). Molecular formula, C_16_H_13_ClN_2_O; molecular weight, 284.74; m. p., 60°C to 62°C.; Rf. value (toluene/ethyl acetate/formic acid, 5:4:1),0.74; I.R. value, 3,415.70(N-H), 2,918.10(C-H str.), 1,618.17(C = O), 1,585.38(N-H bend.), 827.41(C-H bend.). m/e, 285.1, 268.0, 249.0, 223.1, 207.0, 192.0 (100%), 183.1.

***Synthesis of 4-(4-chlorophenyl)-3,4-dihydro-6-phenylpyrimidin-2(1H)-one (5F):*** It was obtained by reaction of compound 3-(4-chlorophenyl)-1-phenylprop-2-en-1-one (3 F) (0.01 M), add 0.01 M of NaOH and urea (0.01 M). Molecular formula, C_16_H_13_ClN_2_O; molecular weight, 284.74; m. p., 104°C to 106°C.; Rf. value (toluene/ethyl acetate/formic acid, 5:4:1), 0.94; I.R. value, 3,442.70(N-H), 3,056.96(C-H str.), 1,635.59(C = O), 1,595.02(N-H bend.), 823.55(C-H bend.).

***Synthesis of 4-(2,4-dichlorophenyl)-3,4-dihydro-6-phenylpyrimidin-2(1H)-one (5G):*** It was obtained by reaction of compound 3-(2,4-dichlorophenyl)-1-phenylprop-2-en-1-one (3G) (0.01 M), add 0.01 M of NaOH and urea (0.01 M). Molecular formula, C_16_H_12_Cl_2_N_2_O; molecular weight, 319.19; m. p., 143°C to 145°C.; Rf. value (toluene/ethyl acetate/formic acid, 5:4:1), 0.88; I.R. value, 3,421.48(N-H), 2,916.17(C-H str.), 1,635.59(C = O), 1,581.52(N-H bend.), 830.65(C-H bend.).

***Synthesis of 4-(2,6-dichlorophenyl)-3,4-dihydro-6-phenylpyrimidin-2(1H)-one (5H):*** It was obtained by reaction of compound 3-(2,6-dichlorophenyl)-1-phenylprop-2-en-1-one (3H) (0.01 M), add 0.01 M of NaOH and urea (0.01 M). Molecular formula, C_16_H_12_Cl_2_N_2_O; molecular weight, 319.19; m. p., 116°C to 118°C.; Rf. value (toluene/ethyl acetate/formic acid, −5:4:1), 0.96; I.R. value, 3,417.63(N-H), 2,918.10(C-H str.), 1,683.74(C = O), 1,585.87(N-H bend.), 850.86(C-H bend.).

***Synthesis of 4-(4-fluorophenyl)-3,4-dihydro-6-phenylpyrimidin-2(1H)-one (5I):*** It was obtained by reaction of compound 3-(2-fluorophenyl)-1-phenylprop-2-en-1-one (3I) (0.01 M), add 0.01 M of NaOH and urea (0.01 M). Molecular formula, C_16_H_13_FN_2_O; molecular weight, 268.29; Rf. value (toluene/ethyl acetate/formic acid, 5:4:1), 0.78; I.R. value, 3,225.05(N-H), 2,920.03(C-H str.), 1,683.74(C = O), 1,596.95(N-H bend.), 815.83(C-H bend.).

***Synthesis of 4-(4-bromophenyl)-3,4-dihydro-6-phenylpyrimidin-2(1H)-one (5K):*** It was obtained by reaction of compound 3-(4-bromophenyl)-1-phenylprop-2-en-1-one (3 K) (0.01 M), add 0.01 M of NaOH and urea (0.01 M). Molecular formula, C_16_H_13_FN_2_O; molecular weight, 268.29; Rf. value (toluene/ethyl acetate/formic acid, −5:4:1), 0.72; I.R. value, 3,415.70(N-H), 2,921.96(C-H str.), 1,677.95(C = O), 1,589.23(N-H bend.), 831.26(C-H bend.).

***Synthesis of 3,4-dihydro-4-(3-nitrophenyl)-6-phenylpyrimidin-2(1H)-one (5M):*** It was obtained by reaction of compound 3-(3-nitrophenyl)-1-phenylprop-2-en-1-one (3 M) (0.01 M), add 0.01 M of NaOH and urea (0.01 M). Molecular formula, C_16_H_13_N_3_O_2_; molecular weight, 295.29; Rf. Value (toluene/ethyl acetate/formic acid, −5:4:1), 0.75; I.R. value, 3,417.63(N-H), 2,916.17(C-H str.), 1,677.95(C = O), 1,596.95(N-H bend.), 831.26(C-H bend.).

***Synthesis of 3,4-dihydro-4-(4-nitrophenyl)-6-phenylpyrimidin-2(1H)-one (5N):*** It was obtained by reaction of compound 3-(4-nitrophenyl)-1-phenylprop-2-en-1-one (3 N) (0.01 M), add 0.01 M of NaOH and urea (0.01 M). Molecular formula, C_16_H_13_N_3_O_2_; molecular weight, 295.29; m. p., 143°C to 145°C.; Rf. value (toluene/ethyl acetate/formic acid, −5:4:1), 0.70; I.R. value, 3,415.70(N-H), 2,918.10(C-H str.), 1,614.31(C = O), 1,598.88(N-H bend.), 696.25(C-H bend.).

***Synthesis of 3,4-dihydro-4-(3,4,5-trimethoxyphenyl)-6-phenylpyrimidin-2(1H)-one (5P):*** It was obtained by reaction of compound 3-(3,4,5-trimethoxyphenyl)-1-phenylprop-2-en-1-one (3P) (0.01 M), add 0.01 M of NaOH and urea (0.01 M). Molecular formula, C_19_H_20_N_2_O_4_; molecular weight, 340.37; m. p., 66°C to 68°C; Rf. value, (toluene/ethyl acetate/formic acid, 5:4:1), 0.82; I.R. value, 3,325.05(N-H), 2,920.03(C-H str.), 1,643.76(C = O), 1,589.23(N-H bend.), 838.98(C-H bend.).

### Synthesis of compounds (6A-6P): general procedure

To a solution of 5A-5P (0.01 mol) in ethanol, β-D-ribofuranose-1,2,3,5-tetra-o-acetate (0.01 mol) was added in the presence of TsOH and the content were refluxed under vacuum with stirring at 155°C to 160°C for 15 to 30 min. The vacuum was removed, and the reaction mixture was protected from moisture by fitting a guard tube. Stirring was further continued for 10 h and vacuum was applied for 10 min. at every hour. The viscous mass thus obtained was dissolved in sodium-containing methanol and boiled for 10 min then left for stirring overnight at room temperature. The reaction mixture was filtered, and the filtrate was evaporated to dryness. The viscous residue, thus obtained was dissolved in ether, filtered, concentrated, and kept in refrigerator overnight to get crystalline product.

***Synthesis of 3,4-dihydro-1-(tetrahydro-3, 4-dihydroxy-5-(hydroxymethyl)furan-2-yl)-4, 6-diphenylpyrimidin-2(1H)-one (6A):*** It was obtained from the reaction of 3,4-dihydro-6-(phenyl)-4-phenylpyrimidin-2(1H)-one (5A) (0.01 mol) in ethanol, β-D-ribofuranose-1,2,3,5-tetra-o-acetate (0.01 mol) was added in the presence of TsOH. Molecular formula, C_23_H_26_N_2_O_7_; molecular weight, 382.41; m. p., 121°C to 123°C; Rf. value (ethyl acetate/n-hexane. 3:7), 0.73; I.R. (KBr cm^−1^), 3,542.99(O-H, str.), 3,286.48(N-H str.), 3,006.82(C-H, str.), 1,613.67 (C = C), 1,380.94 (C = O), 1,114.78 (C-O-C), 744.47(C-H, bend.). 1H-NMR (CDCl_3_-d, δ, ppm), 2.46(s, 1H, NH), 3.22(s, 3H, OH), 3.81 to 3.95(d, 3H, CH_2_, CH), 3.99 to 4.55 (m, 4H, CH), 5.28(d, 1H, CH), 6.41 to 7.259(m, 10H, Ar-CH). m/e, 382.15(M^+^); elemental analysis calculated, C, 65.96; H, 5.80; N, 7.33.

***Synthesis of 3,4-dihydro-1-(tetrahydro-3,4-dihydroxy-5-(hydroxymethyl)furan-2-yl)-6-(2,4-dimethoxyphenyl)-4-phenylpyrimidin-2(1H)-one (6B):*** It was obtained from the reaction of 3,4-dihydro-6-(2,4-dimethoxyphenyl)-4-phenylpyrimidin-2(1H)-one (5B) (0.01 mol) in ethanol, β-D-ribofuranose-1,2,3,5-tetra-o-acetate (0.01 mol) was added in the presence of TsOH. Molecular formula, C_23_H_26_N_2_O_7_; molecular weight, 442.17; m. p., 142°C to 144°C; Rf. value (ethyl acetate/n-hexane, 3:7); 0.80, IR (KBr cm^−1^), 3,440.77(O-H, str.), 3,375.20(N-H, str.), 2,921.96(C-H, str.), 1,504.37 (C = C), 1,215.07 (C = O), 1,193.85 (C-O-C), 815.83(C-H, bend.). 1H-NMR (CDCl3-d, δ, ppm), 2.59(s, 1H, NH), 3.37(s, 3H, OH), 3.54 to 3.61(d, 3H, CH_2_, CH), 3.69(s, 6H, OCH_3_), 4.23 to 4.55 (m, 4H, CH), 5.65 to 5.67(d, 1H, CH), 6.95 to 7.25 (m, 8H, Ar-CH). m/e, 442.2, 417.2, 343.2, 310.0, 288.0, 213.0(100%), 165.1, 135.1. Elemental analysis calculated, C, 62.43; H, 5.92; N, 6.33.

***Synthesis of 3,4-dihydro-1-(tetrahydro-3, 4-dihydroxy-5-(hydroxymethyl)furan-2-yl)-6-(2-methoxyphenyl)-4-phenylpyrimidin-2(1H)-one(6C):*** It was obtained from the reaction of 3,4-dihydro-4-(2-methoxyphenyl)-6-phenylpyrimidine-2(1H)-one(5C) (0.01 mol) in ethanol, β-D-ribofuranose-1,2,3,5-tetra-o-acetate (0.01 mol) was added in the presence of TsOH. Molecular formula, C_22_H_24_N_2_O_6_; molecular weight, 412.5; m. p., 55°C to 57°C; Rf. value (toluene/ethyl acetate/formic acid, 5:4:1), 0.92; I.R.(KBr, cm^−1^), 3,487.06 (O-H, str.), 3,307.69 (N-H, str.), 2,920.03 (C-H, Ar.), 1,596.95 (C = C), 1,091.63 (C-O-C), 827.41(C-H, bend.); 1H-NMR, 1.556(s, 1H, NH), 3.330 to 3.338(s, 3H, OH), 3.343(s, 3H, OCH_3_), 3.740 to 3.75(d, 2H, CH), 4.054 to 4.11(m, 4H, CH), 6.297 to 6.299(d, 2H, CH), 6.902to 7.954(m, 9H, Ar-CH); m/e, 412.23, 362.3(100%), 359.5, 195. Elemental analysis calculated, C, 64.07; H, 5.87; N, 6.79.

***Synthesis of 3,4-dihydro-1-(tetrahydro-3,4-dihydroxy-5-(hydroxymethyl)furan-2-yl)-6-(4-methoxyphenyl)-4-phenylpyrimidin-2(1H)-one (6D):*** It was obtained from the reaction of 3,4-dihydro-6-(4-methoxyphenyl)-4-phenylpyrimidin-2(1H)-one (5D) (0.01 mol) in ethanol, β-D-ribofuranose-1,2,3,5-tetra-o-acetate (0.01 mol) was added in the presence of TsOH. Molecular formula, C_22_H_24_N_2_O_6_; molecular weight, 412.44; m. p., 146°C to 148°C; Rf. value (ethyl acetate/n-hexane, 3:7), 0.62; I.R. (KBr, cm^−1^), 3,375.20 (O-H str.), 3,286.48 (N-H, str.), 2,918.10 (C-H, Ar.), 1,598.88 (C = C), 1,134.07 (C-O-C), 815.83(C-H, bend.); 1H-NMR (CDCl_3_-d, δ, ppm), 2.507(s, 1H, NH), 3.346 (s, 3H, OH), 3.607(s, 3H, OCH_3_), 3.654 to 3.709 (m, 5H, CH,CH_2_), 3.740 to 3.910(d, 3H, CH), 4.111 to 4.13(d, 1H, CH), 6.857-7.038(m, 9H, Ar-CH). m/e, 399.2, 365.1, 305.3, 294.0, 278.0, 249.2, 214.1(100%). Elemental analysis calculated, C, 64.07; H, 5.87; N, 6.79.

**Synthesis of*6-(2-chlorophenyl)-3,4-dihydro-1-(tetrahydro-3, 4-dihydroxy-5-(hydroxymethyl) furan-2-yl)-4-phenylpyrimidin-2(1H)-one (6E):*** It was obtained from the reaction of 4-(2-chlorophenyl)-3, 4-dihydro-6-phenylpyrimidine-2(1H)-one(5E) (0.01 mol) in ethanol, β-D-ribofuranose-1,2,3,5-tetra-o-acetate (0.01 mol) was added in the presence of TsOH. Molecular formula, C_21_H_21_ClN_2_O_5_; molecular weight, 416; m. p., 97°C to 99°C; Rf. value (toluene/ethyl acetate/formic acid, 5:4:1), 0.89; I.R. (KBr, cm^−1^), 3,365.55(O-H, str.), 3,244.05(N-H, str.), 2,850.59(C-H, str.), 1,656.74(C = O), 1,596.95 (C = C), 1,132.14 (C-O-C), 815.83(C-H, bend.), 690.47(C-Cl). 1H-NMR (CDCl_3_-d, δ, ppm), 0.880(d, 1H, CH), 1.254(d, 2H, CH_2_), 2.591(s, 1H, NH), 3.293 to 3.479(s, 3H, OH), 3.899 to 3.982(m, 4H, CH), 6.297(d, 2H, CH), 7.257 to 8.690(m, 9H, Ar-CH). m/e, 416.13, 374.2, 360.2, 359.2 (100%), 258.9; elemental analysis calculated, C, 60.51; H, 5.08; N, 6.72.

***Synthesis of 6-(4-chlorophenyl)-3,4-dihydro-1-(tetrahydro-3,4-dihydroxy-5-(hydroxymethyl) furan -2–yl)-4-phenylpyrimidin-2(1H)-one (6F):*** It was obtained from the reaction of 3,4-dihydro-6-(4-chlorophenyl)-4-phenylpyrimidin-2(1H)-one (5F) (0.01 mol) in ethanol, β-D-ribofuranose-1,2,3,5-tetra-o-acetate (0.01 mol) was added in the presence of TsOH. Molecular formula, C_21_H_21_ClN_2_O_5_; molecular weight, 416.85; m. p., 65°C to 67°C; Rf. value (ethyl acetate/n-hexane, 3:7), 0.61. I.R. (KBr cm^−1^), 3,475.49(O-H, str.), 3,380.98(N-H str.), 2,920.03(C-H, str.), 1,569.95 (C = C), 1,662.52 (C = 0), 1,134.07 (C-O-C), 775.33(C-Cl), 815.83(C-H, bend.). 1H-NMR, 2.034 (s, 1H, NH), 3.034 (s, 3H, OH), 3.325 to 3.412(m, 5H, CH, CH_2_), 3.826 to 3.925(d, 3H, CH), 5.352(d, 1H, CH), 7.256 to 7.561(m, 8H, Ar-CH). m/e, 416.85(M^+^). Elemental analysis calculated, C, 60.51; H, 5.08; N, 6.72.

***Synthesis of 6-(2, 4-dichlorophenyl)-3, 4-dihydro-1-(tetrahydro-3, 4-dihydroxy-5-(hydroxymethyl)furan-2-yl)-4-phenylpyrimidin-2(1H)-one (6G)***: It was obtained from the reaction of (2,4-dichlorophenyl)-3,4-dihydro-6-phenylpyrimidin-2(1H)-one(5G) (0.01 mol) in ethanol, β-D-ribofuranose-1,2,3,5-tetra-o-acetate (0.01 mol) was added in the presence of TsOH. Molecular formula, C_21_H_20_Cl_2_N_2_O_5_; molecular weight, 450.0; m. p., 72°C to 74°C; Rf. value (toluene/ethyl acetate/formic acid, 5:4:1), 0.77; I.R. (KBr, cm^−1^), 3,460.06(O-H str.), 3,175.58(N-H, str.), 3,089.75(C-H, str.), 1,558.38 (C = C), 1,660.60 (C = O), 1,193.85 (C-O-C), 815.85(C-H, bend.), 688.54(C-Cl). 1H-NMR (CDCl_3_-d, δ, ppm), 1.253(s, 1H, CH), 1.286(d, 2H, CH_2_), 2.034(s, 1H, NH), 3.826(s, 3H, OH), 5.352(d, 2H, CH), 7.256 to 7.561(m, 8H, Ar-CH); m/e, 450.2, 387.32, 369.42, 197.17(100%); elemental analysis calculated, C, 55.89; H, 4.47; N, 6.21.

***Synthesis of 6-(2,6-dichlorophenyl)-3,4-dihydro-1-(tetrahydro-3,4-dihydroxy-5-(hydroxymethyl)furan-2-yl)-4-phenylpyrimidin-2(1H)-one (6H):*** It was obtained from the reaction of 3,4-dihydro-6-(2.6-dichlorophenyl)-4-phenylpyrimidin-2(1H)-one (5H) (0.01 mol) in ethanol, β-D-ribofuranose-1,2,3,5-tetra-o-acetate (0.01 mol) was added in the presence of TsOH. Molecular formula, C_21_H_20_Cl_2_N_2_O_5_; molecular weight, 451.3; Rf. value (ethyl acetate/n-hexane,3:7), 0.65; I.R. (KBr cm^−1^), 3,419.56(O-H, str.), 3,274.90(N-H, str.), 3,058.89(C-H, str.), 1,595.02(C = C), 1,681.81 (C = 0), 1,178.43 (C-O-C),775.33(C-Cl), 821.62(C-H, bend.). 1H-NMR (CDCl_3_-d, δ, ppm), 2.800(s, 1H, NH), 3.323 to 3.402(s, 3H, OH), 3.562 to 3.662(d, 3H, CH_2_, CH), 3.677 to 4.685(m, 4H, CH), 5.29 to 5.31(d, 1H, CH), 6.910 to 7.645(m, 8H, Ar-CH). m/e, 451.3(M^+^);elemental analysis calculated, C, 55.89; H, 4.47; N, 6.21.

***Synthesis of 6-(2-fluorophenyl)-3,4-dihydro-1-(tetrahydro-3,4-dihydroxy-5-(hydroxymethyl)furan-2-yl)-4-phenylpyrimidin-2(1H)-one (6I):*** It was obtained from the reaction of 3,4-dihydro-4-(2-flurophenyl)-6-phenylpyrimidine-2(1H)-one(5I) (0.01 mol) in ethanol, β-D-ribofuranose-1,2,3,5-tetra-o-acetate (0.01 mol) was added in the presence of TsOH. Molecular formula, C_21_H_21_FN_2_O_5_; molecular weight, 400.14; m.p.,105°C to 107°C; Rf. value (ethyl acetate/n-hexane, 3:7), 0.52; I.R. (KBr, cm^−1^), 3,363.62(O-H str.), 3,288.40 (N-H str.), 2,921.96 (C-H, Ar.), 1,600.81 (C = C), 1,134.07 (C-O-C), 815.83(C-H, bend.); 1H-NMR (CDCl3-d, δ, ppm), 2.82(s, 1H, NH), 3.33 to 3.41(s, 3H, OH), 3.55 to 3.61(d, 3H, CH_2_, CH), 3.671 to 4.681(m, 4H, CH), 5.51 to 5.31(d, 1H, CH), 7.11 to 7.645(m, 9H, Ar-CH). m/e, 400.14 (M^+^); elemental analysis calculated, C, 62.43; H, 5.92; N, 6.33.

***Synthesis of 6-(4-fluorophenyl)-3,4-dihydro-1-(tetrahydro-3,4-dihydroxy-5-(hydroxymethyl) furan-2-yl)-4-phenylpyrimidin-2(1H)-one (6J)***: It was obtained from the reaction of 3,4-dihydro-4-(2-flurophenyl)-6-phenylpyrimidine-2(1H)-one (5J) (0.01 mol) in ethanol, β-D-ribofuranose-1,2,3,5-tetra-o-acetate (0.01 mol) was added in the presence of TsOH. Molecular formula, C_21_H_21_FN_2_O_5_; molecular weight, 400.14; m. p., 94°C to 96°C; Rf. value (ethyl acetate/n-hexane, 3:7), 0.94; I.R. (KBr, cm^−1^), 3,419.56 (O-H, str.), 3,380.98 (N-H, str.), 2,921.96 (C-H, Ar.), 1,596.95 (C = C), 1,132.14 (C-O-C), 815.83(C-H, bend.); 1H-NMR (CDCl_3_-d, δ, ppm), 2.51(s, 1H, NH), 3.54 (s, 3H, OH), 3.51 to 3.60(d, 3H, CH_2_, CH), 3.661 to 4.181(m, 4H, CH), 5.55 to 5.61(d, 1H, CH), 7.01 to 7.345(m, 9H, Ar-CH). m/e, 400.11(M^+^); elemental analysis calculated, C, 62.43; H, 5.92; N, 6.33.

***Synthesis of 6-(4-bromophenyl)-3,4-dihydro-1-(tetrahydro-3, 4-dihydroxy-5-(hydroxymethyl) furan-2-yl)-4-phenylpyrimidin-2(1H)-one (6K):*** It was obtained from the reaction of 6-(4-bromophenyl)-3,4-dihydro-4-phenylpyrimidin-2(1H)-one (5K) (0.01 mol) in ethanol, β-D-ribofuranose-1,2,3,5-tetra-o-acetate (0.01 mol) was added in the presence of TsOH. Molecular formula, C_21_H_21_BrN_2_O_5_; molecular weight, 461.31; m. p., 68°C to 70°C; Rf. value. (ethyl acetate/n-hexane, 3:7), 0.81; I.R. (KBr, cm^−1^), 3,442.70(O-H, str.), 3,346.27(N-H, str.), 3,064.68 (C-H, str.), 1,604.24(C = C), 1,670.24 (C = 0), 1,174.57 (C-O-C), 640.32(C-Cl), 829.33(C-H, bend.). m/e, 413.3, 381.2, 345.0, 305.3, 249.2, 181.0, 105.0(100%).1H-NMR (CDCl_3_-d, δ, ppm), 2.087(s, 1H, NH), 2.506(s, 3H, OH), 3.357 to 3.399(m, 5H, CH, CH_2_), 3.467 to 3.471(d, 2H, CH), 5.566 (d, 1H, CH), 7.543 to 7.866 (m, 9H, Ar-CH); elemental analysis calculated, C, 54.68; H, 4.59; N, 6.07.

***Synthesis of 3,4-dihydro-1-(tetrahydro-3,4-dihydroxy-5-(hydroxymethyl)furan-2-yl)-6-(3-nitrophenyl)-4-phenylpyrimidin-2(1H)-one (6M):*** It was obtained from the reaction of 3,4-dihydro-6-(3-nitrophenyl)-4-phenylpyrimidin-2(1H)-one (5M) (0.01 mol) in ethanol, β-D-ribofuranose-1,2,3,5-tetra-o-acetate (0.01 mol) was added in the presence of TsOH. Molecular formula, C_21_H_21_N_3_O_7_; molecular weight, 427.41; m.p., 112°C to 114°C; Rf. value (ethyl acetate/n-hexane, 3:7), 0.82; I.R. (KBr cm^−1^), 3,411.84(O-H, str.), 3,319.26 (N-H, str.), 2,918.10(C-H, str.), 1,596.95(C = C), 1,658.69 (C = O), 1,174.57 (C-O-C), 1,386.72(NO2), 796.55(C-H, bend.). 1H-NMR (CDCl_3_-d, δ, ppm), 2.41 (s, 1H, NH), 3.23 (s, 3H, OH), 3.61 to 3.65(d, 3H, CH_2_, CH), 3.66 to 4.29 (m, 4H, CH), 5.51 to 5.61(d, 1H, CH), 7.13 to 7.445 (m, 9H, Ar-CH). Elemental analysis calculated, C, 59.01; H, 4.95; N, 9.83.

***Synthesis of 3,4-dihydro-1-(tetrahydro-3,4-dihydroxy-5-(hydroxymethyl)furan-2-yl)-6-(4-nitrophenyl) -4-phenylpyrimidin-2(1H)-one (6N):*** It was obtained from the reaction of 3,4-dihydro-4-(4-nitrophenyl)-6-phenylpyrimidin-2(1H)-one(5N) (0.01 mol) in ethanol, β-D-ribofuranose-1,2,3,5-tetra-o-acetate (0.01 mol) was added in the presence of TsOH. Molecular formula, C_21_H_21_N_3_O_7_; molecular weight, 427.41; m. p., 96°C to 98°C; Rf. value (toluene/ethyl acetate/formic acid, 5:4:1), 0.89; I.R. (KBr, cm^−1^), 3,448.49(O-H, str.), 3,024.18(N-H, str.), 2,918.10(C-H, str.), 1,602.74(C = O), 1,584.79 (C = C), 1,355.86 (O = N = O), 815.83 (C-H, bend.); 1H-NMR (CDCl_3_-d, δ, ppm), 0.880 (d, 2H, CH_2_), 1.255(s, 1H, CH), 3.321 to 3.703(s, 3H, OH), 3.841 to 3.993(m, 4H, CH), 6.022(d, 2H, CH), 6.724 to 7.947(m, 9H, Ar-CH). m/e, 427.5, 367.0 (100%), 245, 167; elemental analysis calculated, C, 59.01; H, 4.95; N, 9.83.

***Synthesis of 3,4-dihydro-1-(tetrahydro-3,4-dihydroxy-5-(hydroxymethyl)furan-2-yl)-6-(3,4 5-trimethoxyphenyl)-4-phenylpyrimidin-2(1H)-one (6P):*** It was obtained from the reaction of 3,4-dihydro-4-(3, 4, 5-trimethoxyphenyl)-6-phenylpyrimidin-2(1H)-one(5P) (0.01 mol) in ethanol, β-D-ribofuranose-1,2,3,5-tetra-o-acetate (0.01 mol) was added in the presence of TsOH. Molecular formula, C_24_H_28_N_2_O_8_; molecular weight, 472; m. p., 64°C to 66°C; Rf. value (toluene/ethyl acetate/formic acid, 5:4:1), 0.67; I.R. (KBr, cm^−1^), 3,460.06(O-H, str.), 3,175.58(N-H, str.), 3,071.43(C-H, str.), 1,570.80 (C = C), 1,650.13 (C = O), 1,192.89(C-O-C), 810.51(C-H, bend.). 1H-NMR (CDCl_3_-d, δ, ppm), 0.885(s, 9H, CH_3_), 0.878 (s, 1H, CH), 1.253(d, 2H, CH_2_), 2.021(s, 1H, NH), 3.302 to 3.885 (s, 3H, OH), 6.126 (d, 2H, CH), 7.255(m, 7H, Ar-CH); m/e, 472.49, 338.3 (100%), 415.0, 167.07; elemental analysis calculated, C, 61.01; H, 5.97; N, 5.93.

## Results and discussion

The scavenging effects of the synthesized compounds **6A-6P** on the DPPH radical was evaluated according to Leong and Shui et al. Various concentrations (10, 25, 50, 75, 100, and 200 μg/ml) of the test compounds in methanol were added to a 0.1-mM solution of DPPH radical in methanol. All the tests and analysis were undertaken on three replicates and the results averaged. The antioxidant activity of tested compounds revealed that the reaction with DPPH is a time-dependent fashion and the higher the concentration of the tested compounds showed the higher the radical scavenging activity as well as percent inhibition and AAU. However, Compounds **6K, 6F, 6E, 6G, 6H,** and **6M** exhibited potent activity compared by AAU and IC_50_ value. The profiles of the scavenging effect of synthesized compounds are comparable to that of the ascorbic acid as reference compound. The synthesized compounds were compounds **6P, 6D,** and **6M** which exhibited significant antifungal activity that was carried out by cup plate method against fungal strain on strains such as *Candida inconspicua* (MTCC-1074, ATCC16783), *Candida viswanathii* (MTCC-1629, ATCC-22981), *Candida albicans* (MTCC-227, ATCC-10231), *Candida tropicalis* (MTCC-230, ATCC-20336), and *Candida glabrata* (MTCC-3019, ATCC-90030) against standard drug fluconazole. The National Institute of Health, USA, under the drug discovery program of NCI and screened for anticancer activity at a single high dose (10^−5^ M) in full NCI 60 cell lines and the compound 6E, 6N, 6P, 6C showed anticancer activity. The introduction of bromo, chloro, dichloro (at 2, 4 and 2, 6 positions) group showed almost equivalent antioxidant activity as that of the ascorbic acid. Based on the structure activity relationships, it can be concluded that the presence of halogen group and methoxy group at the second, fourth, and sixth position exhibited potent activity.

## Conclusions

A new series of compounds (6A-6P), i.e., pyrimidine analogues, were synthesized by urea and characterized. The synthesized compounds screened for their *in vitro* antioxidant activity by calculating percentage scavenging, AAU and IC_50_ value, antifungal activity, as well as anticancer activity given by the derivative which has chloro, methoxy, nitro, and chloro substitution having furanose contain pyrimidine derivative that showed the most potent activity.

## Authors’ original submitted files for images

Below are the links to the authors’ original submitted files for images. Authors’ original file for figure 1 Authors’ original file for figure 2 Authors’ original file for figure 3 Authors’ original file for figure 4 Authors’ original file for figure 5 Authors’ original file for figure 6 Authors’ original file for figure 7 Authors’ original file for figure 8 Authors’ original file for figure 9 Authors’ original file for figure 10 Authors’ original file for figure 11 Authors’ original file for figure 12 Authors’ original file for figure 13 Authors’ original file for figure 14 Authors’ original file for figure 15
